# Regioselective self-acylating cyclodextrins in organic solvent

**DOI:** 10.1038/srep23740

**Published:** 2016-03-29

**Authors:** Eunae Cho, Deokgyu Yun, Daham Jeong, Jieun Im, Hyunki Kim, Someshwar D. Dindulkar, Youngjin Choi, Seunho Jung

**Affiliations:** 1 Center for Biotechnology Research in UBITA (CBRU), Institute for Ubiquitous Information Technology and Applications (UBITA), Konkuk University, 120 Neungdong-ro, Gwangjin-gu, Seoul 05029, South Korea; 2 Department of Bioscience and Biotechnology, Microbial Carbohydrate Resource Bank (MCRB), Konkuk University, 120 Neungdong-ro, Gwangjin-gu, Seoul 05029, South Korea; 3 Departments of Food Science and Technology, BioChip Research Center, Hoseo University, 20, Hoseo-ro 79beon-gil, Baebang-eup, Asan-si, Chungcheongnam-do, 336-795, Korea

## Abstract

Amphiphilic cyclodextrins have been synthesized with self-acylating reaction using vinyl esters in dimethylformamide. In the present study no base, catalyst, or enzyme was used, and the structural analyses using thin layer chromatography, nuclear magnetic resonance spectroscopy and mass spectrometry show that the cyclodextrin is substituted preferentially by one acyl moiety at the C2 position of the glucose unit, suggesting that cyclodextrin functions as a regioselective catalytic carbohydrate in organic solvent. In the self-acylation, the most acidic OH group at the 2-position and the inclusion complexing ability of cyclodextrin were considered to be significant. The substrate preference was also observed in favor of the long-chain acyl group, which could be attributed to the inclusion ability of cyclodextrin cavity. Furthermore, using the model amphiphilic building block, 2-*O*-mono-lauryl β-cyclodextrin, the self-organized supramolecular architecture with nano-vesicular morphology in water was investigated by fluorescence spectroscopy, dynamic light scattering and transmission electron microscopy. The cavity-type nano-assembled vesicle and the novel synthetic methods for the preparation of mono-acylated cyclodextrin should be of great interest with regard to drug/gene delivery systems, functional surfactants, and carbohydrate derivatization methods.

Cyclodextrins (CDs) are macrocyclic oligosaccharides consisting of six (in α-), seven (in β-), eight (in γ-) or more α-D-(1 → 4) linked glucoses. The structures are shown as a hollow truncated cone shape with the C6 primary hydroxyl groups are located in the narrow rim while the more open rim is crowned by the secondary hydroxyl groups at positions C2 and C3. Based on the torus shape with the hydrophilic exterior and hydrophobic interior, they can incorporate hydrophobic guests in their cavies^1^, and that has drawn interest with regard to enzyme mimetics as well as the potential for use in drug delivery systems. The representative catalytic reaction by CDs is phenyl ester hydrolysis as a model of chymotrypsin, and the reaction mechanism is suggested to involve the rapid association, acyl-intermediate formation and deacylation in alkaline condition^2^. However, the acyl CD product by self-acylation has not yet been reported in organic solvent.

The modification of CDs has widened the application fields for binding receptors^3^,^4^, enzymatic catalysis^5^, and drug carriers^6^,^7^. In the most of the modification studies, various chemical reactions using base catalysts and protecting-groups methods have been reported^8^,^9^, and several steps are required^10^. In addition, since the regioselective substitution of carbohydrates has been a hard task due to their multifunctionality^11^, enzymatic biotransformations are on the rise for amphiphilic acylations^12^,^13^,^14^,^15^. In particular, carbohydrate fatty acid esters have gained attention in food, cosmetic, and pharmaceutical industries as non-toxic and biodegradable surfactants^16^,^17^.

Grafting of hydrophobic moieties onto CDs produces amphiphilic CDs which are self-assembled to nanoarchitectures such as nanoparticles, micelles, and vesicles in aqueous media^18^,^19^,^20^. With the advantage of the cavities within the CDs and the apolar core of the nano-assembly, these amphiphilic CDs are a new generation of CDs that are compatible with bio-membranes. According to the substituted positions and numbers, bouquet-shaped^21^, medusa-like^22^, skirt-shaped^23^, and lollipop style CDs have been developed^24^. Alkyl chains on both, primary, and secondary sides of the CD cavity make bouquet-like, medusa-like, and skirt-shaped derivatives, respectively. When one aliphatic chain is attached to the primary side of CDs, the lollipop structure is expected. The lollipop CDs can also improve the cell targeting of drug-CDs through liposome transport, after insertion in the lipid membrane^25^.

The present work describes the novel ladle-type CDs based on the self-monolaurylated β-CD at the C2 position and the self-assembled nanovesicles. For the self-acylation, the effects of vinyl laurate concentration, temperature, and solvent are presented and discussed. Other vinyl esters (vinyl pivalate, vinyl butyrate, vinyl benzoate, and vinyl stearate) and carbohydrates (glucose, maltodextrin, α-CD, and γ-CD) are also investigated. The ability of 2-*O*-mono-lauryl β-CDs to self-assemble was evaluated using fluorescence spectroscopy, dynamic light scattering and transmission electron spectroscopy.

## Results and Discussion

### Synthesis of mono-2-*O*-lauryl β-CD

**Figure 1:**
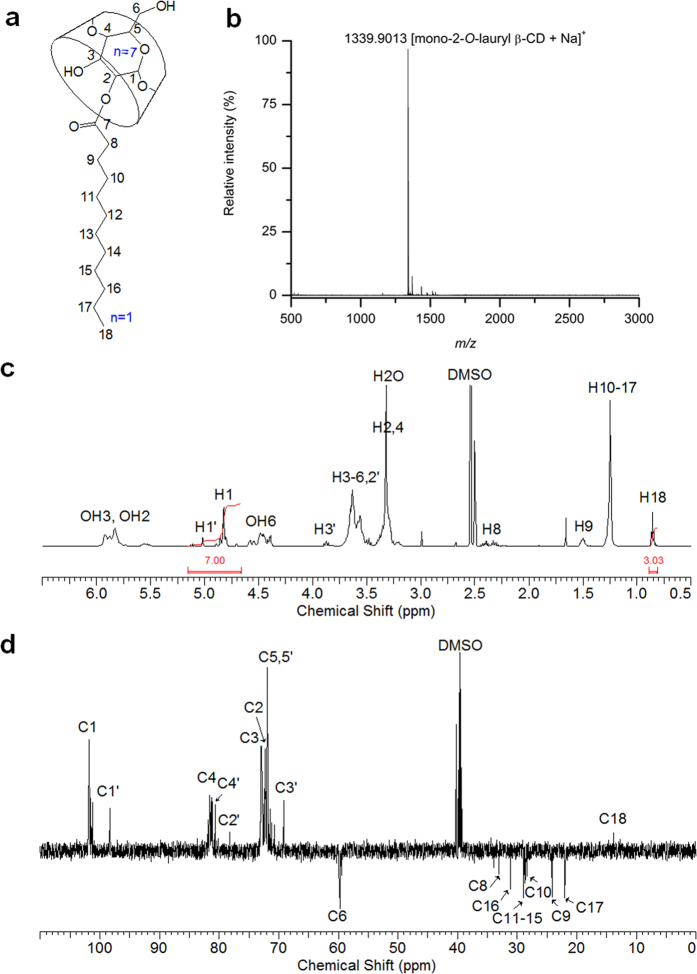
Schematic structure (**a**), MALDI TOF mass spectrum (**b**), ^1^H-NMR spectrum (**c**), and DEPT-135 NMR spectrum (**d**) of mono-2-*O-*lauryl β-CD. Solvent: DMSO-*d*_6_.

Self-acylation of β-CD using vinyl laurate made the ladle-type amphiphilic, mono-2-*O*-lauryl β-CD whose chemical structure is shown in Fig. 1a. After isolation using chromatography on silica gel with elution by 8:5:1 (v/v/v) EtOAc-MeOH-H_2_O, mono-2-*O-*lauryl β-CD was obtained in 17.1% yield and the structure was analyzed by MALDI TOF mass spectrometry and NMR spectroscopy. The pseudo-molecular ion peak at *m/z* = 1339.9013 as [mono-2-*O-*lauryl β-CD+Na]^+^ is shown in its MALDI-TOF mass spectrum (Fig. 1b), and the mono-substitution was also confirmed by integrating the ^1^H-NMR spectrum (Fig. 1c). Other protons were all assigned in the spectrum, and the anomeric protons were separated at around 5.02 and 4.82 ppm by 2-positional substitution. The substitution at the 2 position was clearly shown in DEPT-135 which was used to distinguish between methine and methylene carbons^26^. A large downfield chemical shift of α carbon (C2′, 78.16 ppm), a significant upfield chemical shift of β carbons (C1′ and C3′; 69.17 and 98.30 ppm), and a small upfield chemical shift of γ carbons (C4′) of the acylated glucose unit are observed, while there is no change in the chemical shift of C6 (Fig. 1d). In the ^13^C-NMR spectrum, the carbonyl carbon of ester linkage was also detected at 173.08 ppm, and the ester linkage provides a potential biodegradability in the organism. The structural analyses support the successful regioselective mono-laurylation of β-CD at the C2 position. For effective selective monosubstitution of CDs, a multistep group transfer strategy based on protection/deprotection^8^, complex reaction conditions or precise pH control have been required^27^. Recently, biotransesterification using enzymes has been reported as a mild and simple process compared with conventional chemical catalysis^12^,^15^. Here, we report on the regioselective self-monoacylating CDs without catalyst, enzyme, protection or multi-step reaction.

### Proposed mechanism of self-laurylation on β-CD

**Figure 2:**
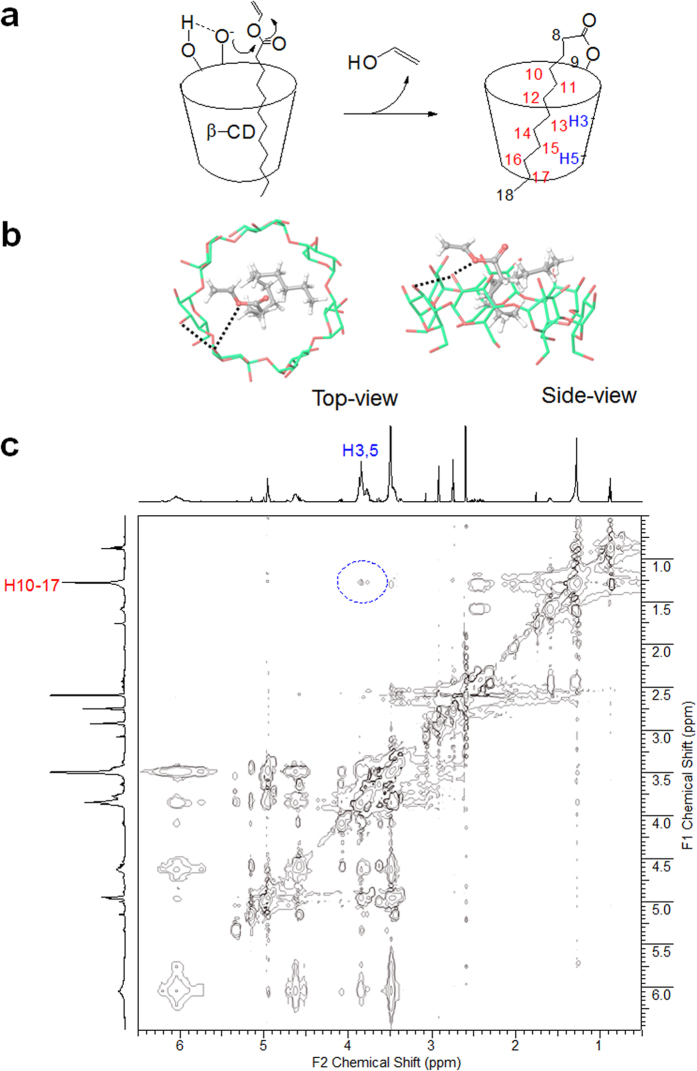
Proposed reaction mechanism (**a**). Side- and top-view for the docked poses of inclusion complex between vinyl laurate and β-CD (**b**). The carbon atoms of vinyl laurate and β-CD were respectively rendered in grey and green colors. The key interaction between vinyl ester and OH3′/OH2 of β-CD is indicated by a dotted line. Contour plot of a ROESY experiment performed on 10 mM mono-2-*O-*lauryl β-CD (500 MHz, 298 K, DMF-*d*_*7*_) showing correlations between protons of the β-CD cavity and of the lauryl chain (**c**).

For the self-laurylation in the basic and polar aprotic solvent, DMF, it is assumed that intramolecular hydrogen bonds between OH3′ and OH2 stabilize the oxyanion at the C2 position of β-CD and the alkyl chain of vinyl laurate is inserted into the cavity (Fig. 2a). Indeed, the C6 hydroxyls, being primary alcohol, is the most basic and often most nucleophilic site, and the hydroxyl group of the C2 position is the most acidic^28^,^29^. A previous molecular dynamic simulations have also suggested the most acidity of OH2 acting as a proton acceptor in the hydrogen bonding between hydroxyl groups at 2 and 3 positions^30^. The reactive oxyanion of the more open rim can undergo a nucleophilic addition reaction to the carbonyl carbon of vinyl ester located closely by the cavity. To evaluate the molecular feasibility of the inclusion complexation between vinyl laurate and β-CD, docking simulation was conducted with the Glide program. The modeling data demonstrate that vinyl laurate is located in the cavity space of β-CD to form a typical inclusion complex (Fig. 2b). The vinyl ester moiety is headed for the secondary rim of the β-CD rather than the primary face. The relatively small vinyl laurate is accommodated in the large cavity by molecular tilting, to minimize dead space between the guest and host during complexation. A representative docking model shows the vinyl ester oxygen of the laurate approaching the oxygen atom at the C2 position in the β-CD. Along with a release of vinyl alcohol after a nucleophilic addition at this point, the mono-2-*O-*lauryl β-CD is produced, and the arrangement for self-acylation also supports the self-inclusion possibility of the product. Figure 2c shows a ROESY spectrum of mono-2-*O-*lauryl β-CD in DMF-*d*_*7*_ with a mixing time of 500 ms, where the interior protons (H3 and H5) of β-CD cavity are correlated with the methylene protons (H10-H17) of the lauryl chain. In the previous study, lollipop-style mono-6-substituted β-CD has also shown the intramolecular complexes by auto-inclusion^24^.

### Effect of the amount of vinyl laurate

**Figure 3:**
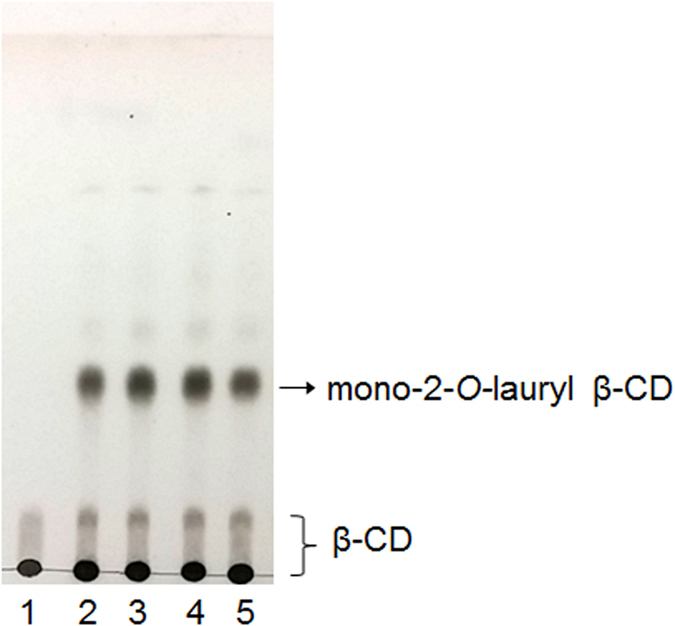
TLC analysis of β-CD reaction mixtures in DMF at 50 °C after 24 h. Mobile phase: BuOH/EtOH/H_2_O (4:2:1, v/v/v). Mole ratio of vinyl laurate/β-CD = 0 (Lane 1); 1 (Lane 2); 2.5 (Lane 3); 5 (Lane 4); 10 (Lane 5).

The mono-laurylation is regarded to be due to the inclusion property of β-CD. Although the ratio of vinyl laurate to β-CD may be increased, the conversion to mono-laurate in 24 h shows no significant difference (Fig. 3). From the TLC densitometric analysis using ImageJ software, the conversions of 41, 44, 46, and 38% were obtained in 1, 2.5, 5, and 10 mole ratios of vinyl laurate/β-CD, respectively. Monosubstitution and the C2 position remained in all cases. Using thermolysin for β-CD esterification, the obtained structure shows a skirt-shaped β-CD containing seven lauryl groups attached to the C2 position, as a major product^12^. This result provides additional evidence for the self-acylation of β-CD via the inclusion of a lauryl group. Since the conventional chemical method of modifying CD at the secondary side involves the activation of OH group by sulfonylation^31^,^32^, cyclic tin intermediate^33^, or deprotonation with sodium hydride in DMF^34^, the present self-acylation can be a useful technique for the C2 regioselective and mono substitution for CDs.

### Effect of temperature

**Figure 4:**
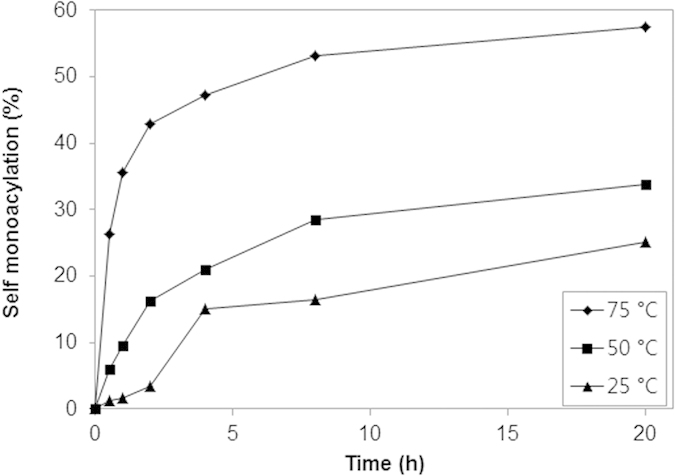
Kinetics of the self-acylation of β-CD with vinyl laurate in DMF. Conditions: 0.09 M β-CD, 0.09 M vinyl laurate, 1 mL DMF, 500 rpm.

The conversion to mono-2-*O-*lauryl β-CD was investigated at different temperatures including 25, 50, and 75 °C. The mono-acylation was found to be increased and then saturated with increasing temperature, and significant conversions (25, 34, and 57% for 25, 50, and 75 °C) were achieved in 20 h (Fig. 4). In general, the increased temperature increases the rate of chemical reactions, due to the increased average velocity and kinetic energy of the reactants causing an increase of the collision frequency and collision fraction between reactants having the minimum energy needed to react. In addition, considering that the pK_a_ of β-CD decreases with increases of temperature^35^, the increased temperature might cause enhanced reactivity of the hydroxyl group in the C2 position for the conversion mono-2-*O-*lauryl β-CD.

### Effect of solvent

**Figure 5:**
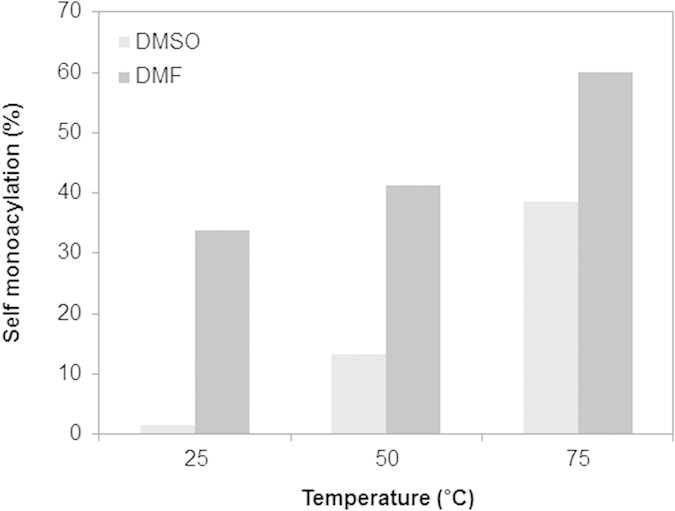
Conversion to mono-2-*O-*lauryl β-CD in DMSO and DMF at 25, 50, and 75 °C.

For the solubility of β-CD, DMF was chosen as a polar aprotic solvent for the present reaction and it was compared with DMSO. Figure 5 shows the tendency of conversion yield in both DMSO and DMF at 25, 50, and 75 °C. The self-monolaurylation occurs with 30–60% in DMF at all temperatures, while the conversion in DMSO shows 0–40%. In water, the present reaction was not observed at 50 °C after 24 h. Since this result is matched with the trend of the SN2 reaction favored solvent, the present self-acylation of β-CD is also suspected to be a nucleophilic addition. Anions are poorly solvated in dipolar aprotic solvents, and as such they are significantly more reactive than the mostly solvated species subject to hydrogen bonding in protic solvents^36^. The self-cleaving ribozymes also have an in-line SN2 reaction using the 2′ hydroxyl group as a nucleophile attacking the phosphodiester and causing the bringing 5′ oxygen to become a leaving group^37^. As another example of the acceleration of a rate by a solvent, the decarboxylation of benzioxazole-3-carboxylic acids were investigated with various solvents, and DMF showed higher reaction rates than DMSO with the least effect being observed in water^38^.

### Vinyl esters and carbohydrates variation

**Figure 6:**
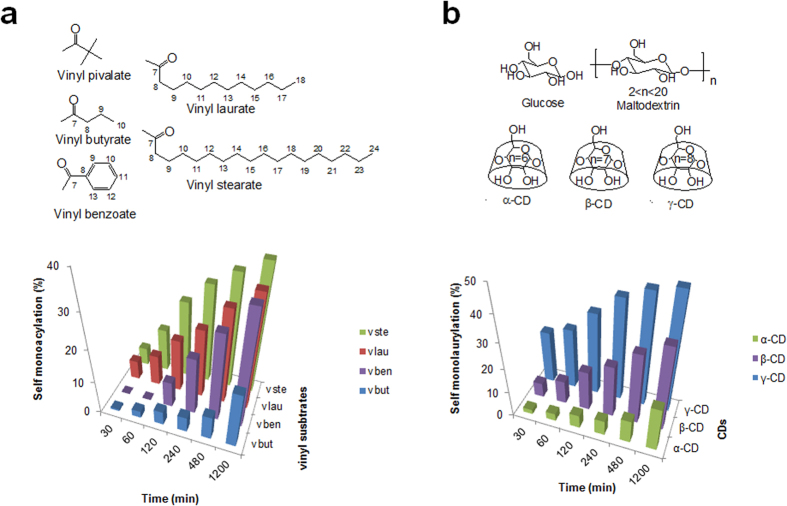
The self-acylation of β-CD with various vinyl esters (**a**) and the self-laurylation with various carbohydrates (**b**) depending on the time period. Insets show the chemical structures of the used vinyl esters and carbohydrates (vbut: vinyl butyrate; vben: vinyl benzoate; vlau: vinyl laurate; vste: vinyl stearate).

To investigate the effect of various acyl groups, β-CD was reacted with vinyl pivalate, vinyl butyrate, vinyl benzoate, vinyl laurate, and vinyl stearate at 50 °C in DMF. The time-dependent reaction aliquot was analyzed, and all vinyl esters except vinyl pivalate produced the mono-acyl β-CD as time goes on (Fig. 6a). Using vinyl butyrate, the amount of conversion was the lowest, and the highest progress was observed in vinyl stearate with 18 carbons. The result might be from the carbon length and inclusion state: the tert-butyl group of vinyl pivalate is too short to be incorporated into the cavity of β-CD; the longer alkyl chain is the better for insertion into the cavity; the benzyl group is preferred for insertion over butyl moiety but the lauryl group precede the benzyl substituent^39^. The benefit of alkyl chain length may be derived from the effective cavity occupation of the folded hydrocarbon chain based on the molecular model (Fig. 2b). Furthermore, the importance of β-CD cavity could be confirmed by the inhibition of self-laurylation in the presence of adamantane carboxylate as a competitive guest which size is almost exactly accommodated by β-CD cavity (Supplementary Information, Fig. S2)^40^.

Considering the reactivity of the 2OH of β-CD, glucose, maltodextrin (linear α-, 1, 4 linked glucans), α- and γ-CD were compared in the same way for self-laurylation. Glucose and maltodextrin show no product, and only the CD makes the acyl product, indicating the significance of cyclic nature for the self-laurylation. Cyclization may induce the characteristic molecular environment that hydrogen bonding networks inside of the ring make the 2OH of glucose more acidic. The order of produced lauryl CD amount is γ-CD > β-CD > α-CD at every time period (Fig. 6b). The oxyanion of O2 is associated with the intramolecular hydrogen bond between 2OH and 3′OH, and it is from the secondary hydroxyl groups of the macrocyclic structure in CDs. The hydrogen bonding belt of the secondary rim is reminiscent of the concerted hydrogen bonding in the catalytic triad of chymotrypsin, where it maintains a relatively unstable negative charge of the serine oxygen for attacking carbonyl carbon in ester hydrolysis^41^. Furthermore, since the mean O2-O3′ distances of γ-, β-, and α-CD are 3.00, 2.86, and 2.81 Å^1^, and the order of pK_a_ values is also γ-CD < β-CD < α-CD^35^,^42^, the best reaction efficiency of self-acylation can be obtained from γ-CD. The available space for the adjustment of substrates in the nucleophilic addition is also expected to favor γ-CD rather than the smaller one (cavity size: 7.5–8.3 Å (γ-CD), 6.0–6.5 Å (β-CD), 4.7–5.3 Å (α-CD)). Taken together, the substrate and carbohydrate preference for self-acylation support the point, the inclusion ability of cavity and the reactivity of O2 induced by cyclic nature of CD.

### Self-organization behavior of mono-2-*O*-lauryl β-CD

**Figure 7:**
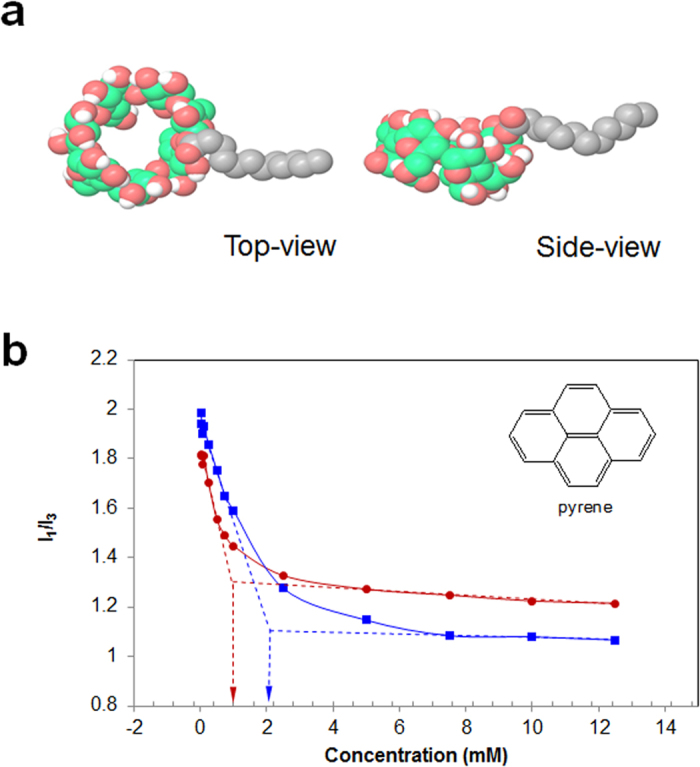
Representative ladle-type images for the molecular model of mono-2-*O*-lauryl β-CD from conformational search tool in the Maestro modeling package (**a**). The relationship of pyrene fluorescence intensity ratios (I_1_/I_3_) with the concentration of mono-2-*O*-lauryl β-CD (red line) and β-CD (blue line) (**b**). The inset shows the chemical structure of pyrene.

The mono-2-*O*-lauryl β-CD is assumed to look like a ladle from the conformational search work in the Maestro software (Fig. 7a). This ladle-type amphiphilic, mono-2-*O*-lauryl β-CD has a stretched-out form in water, and then is self-assembled to nanoaggregates via the van der Waals interaction and hydrogen bonding. The aggregation behavior was monitored by the fluorescence technique with a hydrophobic probe, pyrene. Pyrene is solubilized into the interior of the hydrophobic regions in the presence of micelles or other hydrophobic microdomains in aqueous media^43^. With the emission peaks of pyrene, the intensity ratio of the first to the third peaks (I_1_/I_3_) is regarded as a polarity indicator of the microenvironment around the pyrene. Actually, the I_1_/I_3_ value is 0.58 in hexane and 1.87 in water^44^. Thus, the I_1_/I_3_ value of mono-2-*O*-lauryl β-CD declined with increasing the concentration, and the inflection point of the curve corresponds to the critical aggregation concentration (cac) (Fig. 7b). Since the β-CD cavity provides a hydrophobic microenvironment for entrapped pyrene, the curve of the unmodified β-CD also shows a declining pattern. Interestingly, after lauryl modification of β-CD, the cac values decrease from 2.09 to 0.96 mM, indicating the existence of dual hydrophobic microdomains around the pyrene probe. The pyrene may undergo entry into the β-CD cavity and the self-assembled hydrophobic region. This result suggests that the self-assembled aggregates by mono-2-*O*-lauryl β-CD can be used as effective nanocarrier systems for target hydrophobic substances.

### Size and morphology of the self-assembled architecture

**Figure 8:**
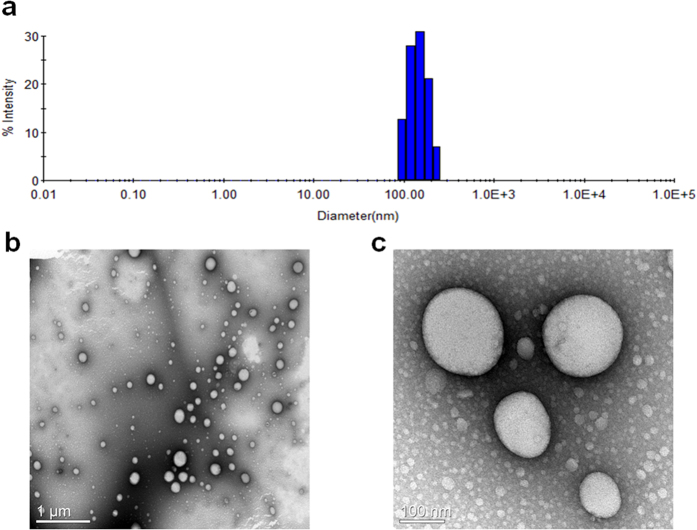
DLS profile (**a**) and TEM images (scale bar: 1 μm (**b**), 100 nm (**c**)) of self-assembled architecture formed by mono-2-*O*-lauryl β-CD.

Self-assembling properties by the amphiphilic β-CD in water were analyzed using the nanoprecipitation method, and investigated by DLS and TEM. The DLS result shows the mean hydrodynamic diameter of the self-assembled architecture as 136 nm with a polydispersity index of 0.07 (Fig. 8a). The DLS data were in good agreement with the microscopic results. The morphology in the aqueous solution was also revealed using TEM, and Fig. 8b shows clear spherical vesicles with diameters of 60–250 nm. The enlarged image also shows small aggregates (5–10 nm), indicating some micellar type aggregates (Fig. 8c). Therefore, we suggest uni- or multilamellar vesicles with 60–250 nm in diameter and micellar aggregates with 5–10 nm in size as the self-assembled architecture of mono-2-*O*-lauryl β-CD (Supplementary Information, Fig. S1). From the molecular model (Fig. 7a), an end-to-end distance for the mono-2-*O*-lauryl β-CD is ca. 2.5 nm and the entire diameter for the micellar form is estimated to be around 6 nm (Maestro, version 2015-3, Schrödinger, LLC, New York, NY, 2015). Skirt-type β-CD with 2–7 decanoyl substituents at the C2 position makes the self-assembled nanoparticle 219 nm in mean diameter^19^. The present ladle-type β-CD may induce a different bilayer organization due to the mono C12 chain and thus the average vesicle size is smaller, where the β-CD/C12/β-CD layer is expected rather than the β-CD/C10/C10/β-CD layer of the previous decanoate β-CD esters. This self-assembling vesicular structure made by the self-acylating β-CD can be applied as cavity-type nanoarchitectures in for drug delivery or functional surfactants.

In the present study, the ladle-type β-CD, mono-2-*O*-lauryl β-CD was successfully synthesized by the self-acylating method using vinyl laurate without a base, catalyst or enzyme. The structure was analyzed with TLC, NMR, and MALDI TOF mass spectrometry. Throughout the investigations on the effects of substrate variations, temperature, solvent choice, and molecular docking, the main factor for self-acylation is considered to be the most acidic 2OH and the inclusion ability of β-CD. Using the amphiphilic hybrid building block, the supramolecular nanovesicles are formed via self-assembly in water. The self-assembling behavior was assessed using fluorescence spectroscopy, DLS and TEM. The present self-acylating and self-assembling β-CD will be a promising material in food, pharmaceutical, and biomedical applications for its nanovesicular architecture, and the self-acylating concept can be applied to the design of novel catalytic cyclic carbohydrates as well as the modification of other carbohydrates using the enhanced reactivity of characteristic hydroxyl groups induced by the presence of cyclic constraint.

## Methods

### Materials

α-D-glucose, maltodextrin, α-CD, vinyl pivalate, vinyl benzoate, pyrene, and dimethylsulfoxide (DMSO) were purchased from Sigma Aldrich. β-CD, γ-CD, vinyl laurate, and vinyl stearate were obtained from Tokyo Chemical Industry Co., Ltd. *N*, *N*-dimethylformamide (DMF, anhydrous 99.8%) was obtained from Alfa Aesar, a Johnson Matthey Company. Organic solvents such as methanol and ethanol were of analytical reagent grade, and the water used was triply distilled. Thin-layer chromatography (TLC) was carried out on Merck Kieselgel 60 F_254_ analytical plates with the specified solvent system. The detection and visualization are followed by spraying 5% sulfuric acid-ethanol and heating for 2 min at 170 °C.

### Self-acylation and product isolation

β-CD (100 mg, 0.09 mmol) was dissolved in 1 mL of DMF, and then vinyl laurate (24 μL, 0.09 mmol) was added. For other self-acylations of β-CD (100 mg, 0.09 mmol), vinyl pivalate (13.7 μL, 0.09 mmol), vinyl butyrate (11.7 μL, 0.09 mmol), vinyl benzoate (12.7 μL, 0.09 mmol), and vinyl stearate (28 mg, 0.09 mmol) were used. For self-laurylation with other carbohydrates, α-CD (85.6 mg, 0.09 mmol), γ-CD (114 mg, 0.09 mmol), glucose (15.8 mg, 0.09 mmol) and maltodextrin (96.3 mg, 0.09 mmol) were used. After stirring at 50 °C for 24 h, the product was isolated by chromatography on silica gel with 8:5:2 or 8:5:1 (v/v) EtOAc-MeOH-H_2_O as eluent^15^. For time course data, every 1 μL from reaction mixture was quenched and analyzed at 0.5, 1, 2, 4, 8, and 20 h. The progress of reactions was analyzed by TLC densitometric analysis using NIH ImageJ software.

### Mono-2-*O*-butyryl β-cyclodextrin

^1^H NMR (600 MHz, DMSO-*d6*): δ 5.92-5.52 (br, O(2)H, O(3)H), 5.03 (d, 1H, H1′), 4.90-4.71 (m, 6H, H1), 4.63-4.41 (br, O(6)H), 3.89 (t, 1H, H3′), 3.66-3.22 (m, H2-6), 3.18 (d, 1H, H3′), 2.44-2.29 (m, 2H, H8), 1.58-1.51 (m, 2H, H9), 0.90 (t, 3H, H10); ^13^C NMR (600 MHz, DMSO-*d6*): δ 173.06 (C7), 101.98 (C1), 98.49 (C1′), 81.56 (C4), 80.84 (C4′), 78.23 (C2′), 73.12 (C3), 72.46 (C2), 72.09 (C5), 69.38 (C3′), 59.96 (C6), 35.13 (C8), 17.68 (C9), 13.55 (C10); MALDI-TOF MS: 1227.6432 [mono-2-*O*-butyryl β-cyclodextrin+Na]^+^.

### Mono-2-*O*-benzoyl β-cyclodextrin

^1^H NMR (600 MHz, DMSO-*d6*): δ 8.10-7.96 (d, 2H, H9, 13), 7.64-7.58 (t, 1H, H11), 7.52-7.46 (t, 2H, H10, 12), 6.03-5.70 (br, O(2)H, O(3)H), 5.20 (d, 1H, H1′), 4.94-4.77 (m, 6H, H1), 4.68-4.45 (br, O(6)H), 4.12 (t, 1H, H2′) 3.70-3.22 (m, H2-6), 3.17 (d, 1H, H3′); ^13^C NMR (600 MHz, DMSO-*d6*): δ 165.97 (C7), 133.08 (C11), 131.29 (C8), 129.81, 129.50 (C9, 13), 128.44, 128.15 (C10, 12), 102.00 (C1), 98.20 (C1′), 81.62 (C4), 80.92 (C4′), 78.11 (C2′), 73.03 (C3), 72.54 (C2), 72.07 (C5), 69.67 (C3′), 59.97 (C6); MALDI-TOF MS: 1261.6154 [mono-2-*O*-benzoyl β-cyclodextrin+Na]^+^.

### Mono-2-*O*-stearyl β-cyclodextrin

^1^H NMR (600 MHz, DMSO-*d6*): δ 6.60-6.10 (br, O(2)H, O(3)H), 5.01 (d, 1H, H1′), 4.88-4.69 (m, 6H, H1), 4.64-4.38 (br, O(6)H), 3.87 (t, 1H, H3′), 3.63-3.20 (m, H2-6), 2.44-2.26 (m, 2H, H8), 1.49 (m, 2H, H9), 1.23 (s, 26H, H10-23), 0.85 (m, 3H, H24); ^13^C NMR (600 MHz, DMSO-*d6*): δ 166.23 (C7), 102.23 (C1), 98.53 (C1′), 81.69 (C4), 80.96 (C4′), 78.38 (C2′), 73.12 (C3), 72.46 (C2), 72.09 (C5), 69.37 (C3′), 59.96 (C6), 33.25 (C8), 31.36 (C22), 29.12-28.53 (C10-21), 24.74 (C9), 22.16 (C23), 14.04 (C24); MALDI-TOF MS: 1423.9385 [mono-2-*O*-stearyl β-cyclodextrin+Na]^+^.

### Mono-2-*O*-lauryl α-cyclodextrin

^1^H NMR (600 MHz, DMSO-*d6*): δ 5.84-5.41 (br, O(2)H, O(3)H), 4.97 (d, 1H, H1′), 4.85-4.67 (m, 5H, H1), 4.65-4.44 (br, O(6)H), 4.00 (t, 1H, H3′), 3.64-3.54 (m, H2′, 3-6), 3.43-3.27 (m, H2, 4), 2.42-2.29 (m, 2H, H8), 1.50 (m, 2H, H9), 1.25 (s, 18H, H10-17), 0.86 (m, 3H, H18); ^13^C NMR (600 MHz, DMSO-*d6*): δ 162.99 (C7), 102.06 (C1), 98.80 (C1′), 82.30 (C4), 81.92 (C4′), 79.40 (C2′), 73.34 (C3), 72.58 (C2), 72.15 (C5), 69.62 (C3′), 60.06 (C6), 35.84 (C8), 31.42 (C16), 29.09-28.68 (C10-15), 24.37 (C9), 22.22 (C17), 14.06 (C18); MALDI-TOF MS: 1177.6322 [mono-2-*O*-lauryl α-cyclodextrin+Na]^+^.

### Mono-2-*O*-lauryl γ-cyclodextrin

^1^H NMR (600 MHz, DMSO-*d6*): δ 6.82-6.18 (br, O(2)H, O(3)H), 5.16 (d, 1H, H1′), 5.11-4.77 (m, 7H, H1), 4.75-4.45 (br, O(6)H), 3.80 (t, 1H, H3′), 3.69-3.16 (m, H2-6, 2′) 2.46-2.28 (m, 2H, H8), 1.50 (m, 2H, H9), 1.25 (s, 15H, H10-17), 0.85 (m, 3H, H18); ^13^C NMR (600 MHz, DMSO-*d6*): δ 166.29 (C7), 101.56 (C1), 97.67 (C1′), 81.35 (C4), 80.75 (C4′), 78.94 (C2′), 73.22 (C3), 72.84 (C2), 72.18 (C5), 69.85 (C3′), 59.89 (C6), 33.29 (C8), 31.34 (C16), 29.09-28.57 (C10-15), 24.32 (C9), 22.14 (C17), 14.04 (C18); MALDI-TOF MS: 1501.8729 [mono-2-*O*- lauryl γ-cyclodextrin+Na]^+^.

### Nuclear magnetic resonance (NMR) spectroscopy

A Bruker Avance 600 MHz spectrometer was used to record the ^1^H-NMR, ^13^C-NMR, and DEPT spectra. The NMR analyses were performed in DMSO-*d6* at room temperature. ROESY data was recorded with 256/2048 data points using Bruker Avance 500 MHz spectrometer in DMF-*d*_*7*_ at room temperature.

### Matrix-assisted desorption/ionization time-of-flight mass spectrometry (MALDI-TOF MS)

The mass spectrum was obtained using a MALDI-TOF mass spectrometer (Voyager-DE^TM^ STR BioSpectrometry, PerSeptive Biosystems, Framingham, MA, USA) using the positive-ion mode. 2, 5-Dihydroxybenzoic acid (DHB) was used as the matrix.

### Computational method

Starting coordinates for the β-CD were obtained from the Protein Data Bank (PDB id. 3CGT) and vinyl laurate was built using Maestro software (ver. 2015-3, Schrodinger Inc.). Molecular docking simulations were performed with the vinyl laurate onto the β-CD under the ligand-flexible mode using the Glide docking module^45^. The molecular grid was established using the receptor grid generation tool in a 20 Å sized cubic box, to estimate potential energy of the system. To archive docking accuracy, the extra precision *XP* mode with Glide *XP* scoring function was applied during the ranking process for the docked poses. The structural model for the mono-2-O-lauryl β-CD in water was calculated by Advanced Conformation Search module in the Maestro package under the mixed torsional/low-mode sampling mode. During the conformation search, the GB/SA implicit model with the OPLS2005 force field was used.

### Fluorescence spectroscopy

For the fluorescence measurement, mono-2-*O*-lauryl β-CD or unmodified β-CD was prepared with concentrations of 0.05 to 12.5 mM in water. A small amount of a hydrophobic probe, pyrene solution in acetone, was added into each sample giving a final concentration of 1 μM in solution. Acetone was not removed, and its final content was 0.1%, v/v^46^. All samples were sonicated for 15 min and mixed for 15 h at 26 °C before measurement. Fluorescence emission spectra of pyrene were recorded using a fluorescence spectrophotometer (SIMADZU, RF-5310PC) at room temperature. The probe was excited at 335 nm, and the emission spectra were obtained in the range of 350–500 nm^47^.

### Nanoprecipitation technique

Using the nanoprecipitation technique^48^, 1 mL of mono-2-*O*-lauryl β-CD (1.25 mM) dissolved in ethanol is dropped slowly through a syringe into water (1 mL) and subjected to magnetic stirring. The nanospheres were formed immediately, and the colloidal suspension obtained was submitted to evaporation under reduced pressure to remove ethanol. The resulting colloidal suspension was stored in closed vials at room temperature.

### Dynamic light scattering (DLS)

The resulting colloid was measured using a Wyatt Technology DynaPro Plate Reader for the assessing the nanoarchitecture size distribution of the mono-2-*O*-lauryl β-CD.

### Transmission electron microscopy (TEM)

The colloidal suspension by mono-2-*O*-lauryl β-CD was loaded onto a Formvar-coated copper grid (200 mesh) and air-dried. For negative staining, 2% uranyl acetate solution was used. Then, the self-assembled nanoarchitecture was examined using energy-filtering TEM (LIBRA 120, Carl Zeiss, Germany).

## Additional Information

**How to cite this article**: Cho, E. *et al.* Regioselective self-acylating cyclodextrins in organic solvent. *Sci. Rep.* **6**, 23740; doi: 10.1038/srep23740 (2016).

## Supplementary Material

Supplementary Information
