# Supplementary material for: Regioselective self-acylating cyclodextrins in organic solvent
Source: Sci Rep. 2016 Mar 29;6:23740. doi: 10.1038/srep23740 (PMC4810458; doi:10.1038/srep23740)
Supplement: Supplementary Information [file srep23740-s1.pdf]

Supplementary information

Regioselective self-acylating cyclodextrins in organic solvent

Eunae Cho,[†] *Deokgyu Yun,*[‡] *Daham Jeong,*[‡] *Jieun Im,*[‡] *Hyunki Kim,*[‡] *Someshwar D Dindulkar,*[†] *Youngjin Choi,*[§] *Seunho Jung*,*[†,‡]

[†]Center for Biotechnology Research in UBITA (CBRU), Institute for Ubiquitous Information Technology and Applications (UBITA), Konkuk University, 120 Neungdong-ro, Gwangjin-gu, Seoul 05029

[‡]Department of Bioscience and Biotechnology, Microbial Carbohydrate Resource Bank (MCRB), Konkuk University, 120 Neungdong-ro, Gwangjin-gu, Seoul 05029, South Korea

[§] Departments of Food Science and Technology, BioChip Research Center,Hoseo University, 20, Hoseo-ro 79beon-gil, Baebang-eup, Asan-si, Chungcheongnam-do, 336-795, Korea

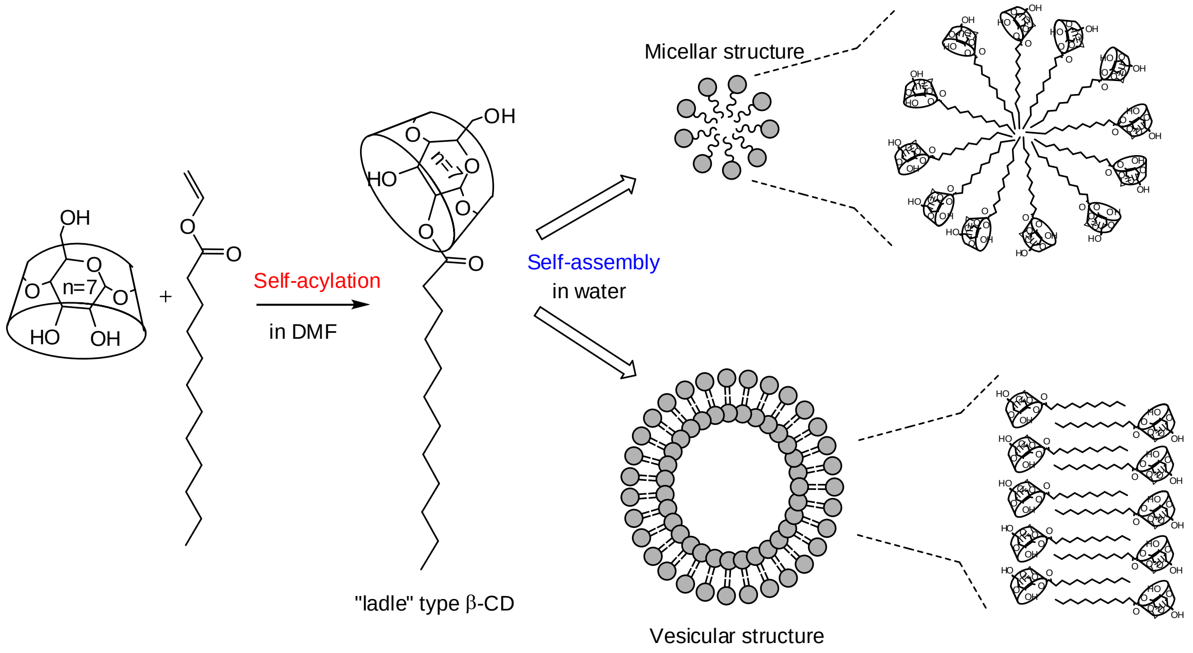

Figure S1. Regioselective self-acylating and self-assembling mono-2-*O*-lauryl β-CD.

The effect of a competitor (adamantane carboxylate) on self-laurylation of β-CD. β-CD (50 mg, 0.044 mmol) was dissolved in 0.5 mL of DMF, and then 1-adamantanecarboxylic acid (7.9 mg, 0.044 mmol or 79 mg 0.44 mmol) was added. After stirring at 50 ℃ for 3 h, vinyl laurate (12 μL, 0.044 mmol) was added and reacted for 24 h.

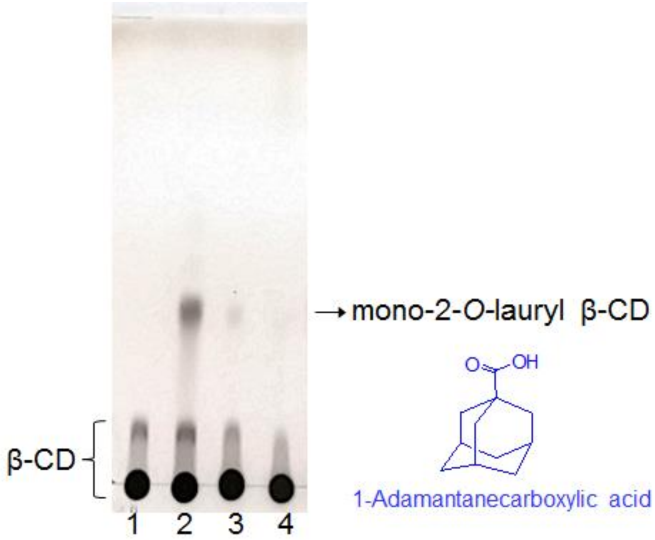

Figure S2. TLC analysis of β-CD reaction mixtures in DMF at 50 ℃ after 24 h. Mobile phase: BuOH/EtOH/H_2O (4:2:1, v/v/v). β-CD (Lane 1); β-CD/vinyl laurate (mole ratio= 1/1) (Lane 2); β-CD/vinyl laurate/1-adamantanecarboxylic acid (mole ratio= 1/1/1) (Lane 3); β-CD/vinyl laurate/1-adamantanecarboxylic acid (mole ratio= 1/1/10) (Lane 4).
